# The Psychosocial Impact of Cleft Lip and/or Palate on Caregivers and Individuals in Low- and Middle-Income Countries: A Narrative Review

**DOI:** 10.1177/10556656251343393

**Published:** 2025-06-16

**Authors:** Nicola M. Stock, Bruna Costa, Anna Zarola, Allyn Auslander, Hugh Brewster, Phyllida Swift, Leela Imam, Usama Hamdan, Gareth Davies, Sara Horne, Priti P. Desai

**Affiliations:** 1 Centre for Appearance Research, 1981 University of the West of England, Bristol, UK; 2 Operation Smile, Virginia Beach, Virginia, USA; 3 Transforming Faces, Toronto, Ontario, Canada; 4 Face Equality International, London, UK; 5 Smile Train, Bengaluru, India; 6 Global Smile Foundation, Westwood, MA, USA; 7 507034 European Cleft Organisation, Perpignan, France; 8 Director of Global Speech and Hearing Programs, Smile Train, Fort Lauderdale, FL, USA; 9 3627 East Carolina University, Greenville, NC, USA

**Keywords:** LMIC, low-income, middle-income, psychosocial adjustment, quality of life, cleft lip and palate

## Abstract

Low- and middle-income countries (LMICs) represent the highest incidence, morbidity, and Disability Adjusted Life Years for cleft lip and/or palate (CL/P) globally. Most cleft care models now include psychosocial support, yet the psychosocial impacts of CL/P in LMICs are understudied. This narrative review aimed to synthesize published literature to provide a foundation for future research and evidence-based psychosocial support. Seventy-one peer-reviewed articles published between September 2003 and July 2024 were included. Themes pertaining to caregivers included Social Experiences, Emotional Well-Being, Treatment Experiences, and overall Quality of Life. Themes regarding individuals with CL/P included Developmental Trajectory, Social Integration, Emotional Health, Satisfaction with Treatment, and Health-Related Quality of Life. Most articles focused on upper-middle-income countries. East Asia and Pacific was the most studied geographical region in LMICs, while Europe and Central Asia was the least studied. Only 28 of 135 LMICs (20.7%) were represented in the included psychosocial literature. Most studies (69%) utilized a quantitative design. Sample sizes ranged from 2 to 295, with 73% of studies recruiting from a single site. Few studies (28%) compared their data to reference or control groups. Just over half of studies used standardized, validated measures. Most studies were led by LMIC teams. This review calls for ongoing monitoring of psychological health in individuals with CL/P and their caregivers and coordinated investment into multifaceted psychosocial program in LMICs. Building practice-relevant research capacity in LMICs to develop the evidence-base is essential.

## Introduction

Each year, approximately 1 in every 730 infants are born with a cleft lip and/or palate (CL/P) in the most resource-deprived parts of the world.^1^ Countries are defined as low- or middle-income (LMICs) by the World Bank according to Gross National Income per capita.^2^ Investigations into the global burden of CL/P have shown the highest death and morbidity rates to be in LMICs.^1,3^ The impact of CL/P on Disability Adjusted Life Years, a summary measure of the years lived with disability and the years of life lost, has also been found to be far higher in countries with low levels of resource,^3^ although this figure may still be underestimated.^4^ Low- and middle-income countries are not uniform, however, and further investigation of the burden of CL/P according to geographical region, classification, and cultural context is needed.

In supporting global health agendas, which emphasize the importance of equal access to high-quality health care without financial burden, nongovernmental philanthropic organizations (NGOs) contribute much of the funding for CL/P care in LMICs.^5^ Recent years have observed a shift away from the “surgical mission” model to one that is more holistic and locally sustainable.^6,7^ Alongside other core disciplines, this focus on comprehensive cleft care has highlighted the need for integrated psychosocial support for individuals and families with CL/P in LMICs. In the last two decades, recognition of the psychological and social impacts of CL/P has grown considerably.^8^ Research conducted in high-income countries (HICs) has documented emotional, social, educational, and treatment-related concerns in young people and adults born with CL/P,^9^ as well as a broader impact on the well-being of caregivers and families.^10^ These findings are now reflected in international treatment guidelines, whereby cleft teams are typically required to include access to a clinical psychologist or other psychosocial provider.^6,11^

While less is known about psychological outcomes regarding CL/P in LMICs, several studies have examined the broader social environment. For example, in a study of 200 pregnant women in Nigeria, approximately half of participants reported to have knowledge of CL/P, yet only 19.8% were able to correctly identify the condition.^12^ In a second Nigerian study of 650 men and women from the general population,^13^ many attributed the etiology of CL/P to “God's will,” “supernatural” phenomena, sickness or infection, or undesirable behaviors by the mother. A recent systematic review^14^ found cultural beliefs to be the main cause of misconceptions surrounding CL/P. In turn, such misconceptions may lead to a range of damaging consequences, including infanticide, a lack of or inappropriate treatment, stigmatization, and social exclusion.^14–16^

Recent years have seen an increase in published articles relating to the psychosocial impact of CL/P in LMICs. The aim of this narrative review was to synthesize the literature published on the psychosocial impact of CL/P on children, adolescents, adults, and their caregivers living in LMICs, with the goal of providing a foundation for future research and evidence-based psychosocial support across cultural and economic contexts.

## Methods

### Inclusion Criteria

All original, peer-reviewed articles pertaining to the psychological adjustment of individuals with CL/P and caregivers of children with CL/P were included. In line with other recent reviews, articles were included if published from January 1, 1980, onward. Articles published up to July 31, 2024, were included in this review. Quantitative, qualitative, and mixed-methods articles were considered. Articles relating to all types of syndromic and nonsyndromic CL/P were included. No age restrictions for individuals or parents were enforced. Articles published online while “in press” were also included where the full article was available. Articles published in all languages were included where necessary and where English translations could be reliably obtained.

### Exclusion Criteria

Case studies, protocol papers, and unpublished dissertations were excluded. Articles relating to “visible difference,” “disfigurement,” other craniofacial conditions (such as craniosynostosis) were excluded. Articles were also excluded if the sample included a mix of CL/P and other conditions, and the results were not separated according to condition. Articles describing findings from HICs (as defined by the World Bank classifications in 20242) were excluded. No literature reviews, systematic reviews, summary articles, book chapters, reports, or meta-analyses published during the search period were included but were stored separately for reference.

### Search Strategy

**Figure 1.**
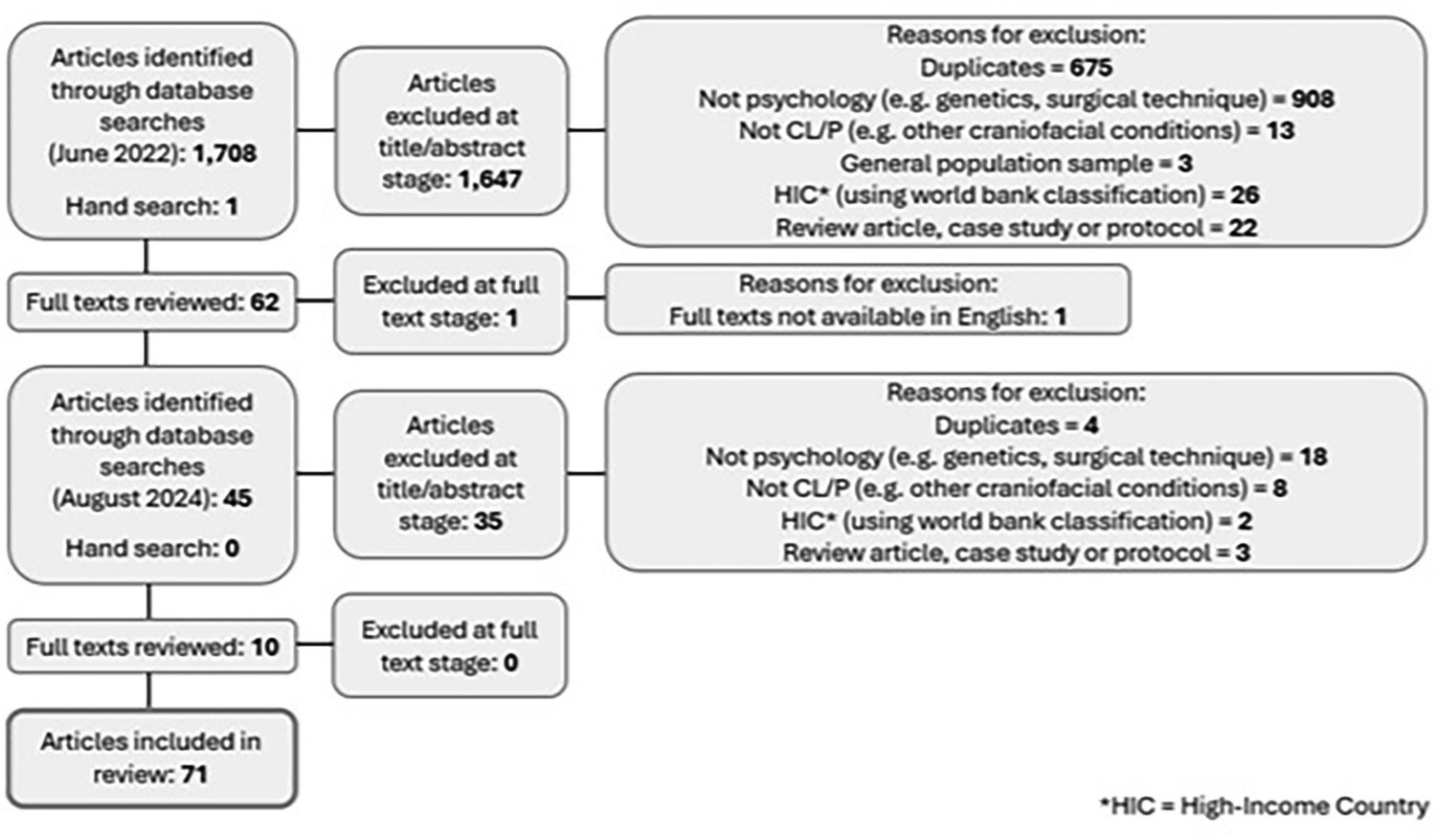
Preferred Reporting Items for Systematic Reviews and Meta-Analyses (PRISMA) flow diagram.

Databases included PsycInfo, MEDLINE, Science Direct, CINAHL Plus, and Scopus. Search terms identified within the article title, abstract, or keywords are presented in Supplemental Table 1. The reference lists of previous relevant reviews were hand-checked to reduce the likelihood of any abstracts being missed. Any duplicates were removed. The initial searches were run in June 2022 and identified 61 eligible full texts for inclusion. These searches were updated in August 2024 and a further 10 eligible articles were included. Titles and abstracts were screened by two independent reviewers. To assess quality control, 40% of abstracts were double screened. The agreement rating was 94.3% (Cohen kappa: 0.76). Any minor discrepancies were discussed until full agreement was reached. Full texts were then screened by the first author (Figure 1). Data regarding methodological details (such as country, classification, study design, sample description and measures used) were extracted for each included paper, alongside key findings. Key findings were grouped together according to primary topic and summarized in relation to novel themes and subthemes. Data extraction was completed by two reviewers and cross-checked by a third reviewer for accuracy.

A quality assessment of each article was not conducted due to the current lack of appropriate tools for use in narrative reviews. The Preferred Reporting Items for Systematic Reviews and Meta-Analyses Checklist^17^ was followed where applicable to ensure the quality of reporting.

## Results

A total of 71 articles were considered eligible for inclusion in this review. Including the two studies that included multiple countries, 41 studies (57.0%) reported on upper-middle-income countries, 26 studies (36.1%) reported on lower-middle-income countries and 5 studies (6.9%) reported on low-income countries. Geographical regions were represented as follows: East Asia and Pacific (n = 23; Cambodia, China, Indonesia, Laos, Malaysia, the Philippines, Thailand, Vietnam); Europe and Central Asia (n = 4; Turkey); Latin America and the Caribbean (n = 15; Argentina, Brazil, Colombia, El Salvador, Honduras, Peru); Middle East and North Africa (n = 9; Egypt, Iran, Jordan, Syria); South Asia (n = 12; India, Pakistan); and Sub-Saharan Africa (n = 17; Benin, Ethiopia, Ghana, Kenya, Nigeria, South Africa, Uganda, Zimbabwe). The most represented geographical regions were East Asia and Pacific (30.0%) and Sub-Saharan Africa (23.9%). The least represented regions were Middle East and North Africa (11.3%) and Europe and Central Asia (5.6%). The most frequently studied countries were India (n = 10), Brazil (n = 8), and Thailand (n = 7).

Included studies were published from 2003 onwards, with a broad trend of an increase in publications over time. Forty-nine studies (69%) used a quantitative design, 17 (24%) utilized a qualitative design, and 5 (7%) used a mixed-methods design. The most common methodology was a cross-sectional questionnaire-based design, administered in either a written or interview-style format (n = 40, 56%). Sample sizes for quantitative studies ranged from 2 to 295 participants, with a cumulative total for all studies of 5795. Fifteen of the 49 quantitative studies (31%) had an n < 50. Participants in most studies (n = 46, 65%) were caregivers. Ten studies reported on individuals with CL/P across a wide age range (0-75 years, 14%). Six studies (8%) included both individuals and their caregivers, while five studies (7%) only included children, and four studies (6%) focused on adults. Most studies (n = 52, 73%) recruited from a single site. Twenty studies (28%) included a control or reference group. Thirty-seven studies (52%) utilized standardized, validated measures. Fifty-one studies (72%) were carried out by a full authorship team from an LMIC, while a further 7 studies (10%) represented a collaboration with a HIC but were led by an LMIC. Six studies (8%) were a collaboration led by a HIC, and seven studies (10%) were conducted by HICs with no LMIC collaborators.

Of the 71 included articles, 34 (48%) pertained to the psychological health of the caregiver, 22 (31%) discussed the psychological impact on the individual, and 15 (21%) included both caregivers and individuals. Caregiver and individual results are reported separately. When engaging with the Results, readers are encouraged to consider the findings within the relevant geographical and cultural contexts and with reference to the methodological characteristics of each included article (Table 1).

**Table 1.** An Overview of the Articles Included in This Review Categorized by Geographical Region.

Country (income category)^a^	Author and year	Aim(s)	Design	Recruitment site(s)	Sample	Reference group(s)	Measure(s)	Theme(s)
East Asia and Pacific								
China (Upper-middle income)	Ha et al, 2013^78^	To obtain descriptive information of behavioral patterns in school-age children with CL/P	Quantitative, cross-sectional, questionnaire	Single hospital	93 children with CL/P aged 6-11 years	US normative data and 100 children without CL/P	Child Behavior Checklist	INDIVIDUALS: Developmental Trajectory, Social Integration
	Lin et al, 2020^82^	To assess oral health-related quality of life in children with CL/P	Quantitative, cross-sectional, questionnaire	Single hospital	120 children and adolescents (49 female, 71 male) with CL/P aged 8-15 years and their parents	None	Chinese version of the Child Oral Health Impact Profile	INDIVIDUALS: Emotional Health, Health-Related Quality of Life
	Yuan et al, 2022^54^	To explore and compare the level and potential influential factors associated with resilience among parents of children with CL/P	Quantitative, cross-sectional, questionnaire	Two centers	248 parents (179 mothers and 69 fathers) of children with CL/P aged 1-13 years	None	Chinese versions of Resilience Scale-14, Herth Hope Index, Multidimensional Scale of Perceived Social Support, Revised Life Orientation Test, Parenting Stress Index-Short Form and Coping Health Inventory for Parents	CAREGIVERS: Emotional Well-Being
Indonesia (Upper-middle income)	Hasanuddin et al, 2024^22^	Investigate how parents’ sociocultural backgrounds impact cleft management	Mixed methods, interview based on a questionnaire, focus groups	Six tertiary care hospitals during a medical mission	202 parents (141 mothers and 61 fathers) of children and members of 10 medical teams	None	Nonvalidated questionnaire previously developed by Naram (2013), translated into Indonesian	CAREGIVERS: Social Experiences, Treatment Experiences
	Soedjana et al, 2022^79^	To evaluate psychosocial problems in school-age patients with CL±P after one or more surgical interventions	Quantitative, cross-sectional, questionnaire	Single center	104 children (57% male) with CL±P, median age of 8 years	Original normative data	Child Behavior Checklist	INDIVIDUALS: Developmental Trajectory, Social Integration
	Soeselo et al, 2019^56^	To identify parents’ knowledge, attitudes and behavior towards CL/P	Quantitative, cross-sectional, questionnaire	Medical mission at single hospital	26 parents of infants with CL/P aged 0-1 years old	None	Nonvalidated questionnaire	CAREGIVERS: Treatment Experiences
Laos (Lower-middle income)	Prathanee et al, 2011^85^	To explore satisfaction with speech and treatment outcomes, alongside treatment needs	Quantitative, cross-sectional questionnaire completed via interview	Single hospital	9 caregivers of children aged 0-2 years, 9 caregivers of older children, 9 children aged 8+ years	N/A	Nonvalidated 5-point Likert questionnaire	INDIVIDUALS: Satisfaction with Treatment
Malaysia (Upper-middle income)	Goh et al, 2019^75^	To describe the audiology status and behavioral patterns of school-aged children with CL/P	Quantitative, cross-sectional, questionnaire	Single university hospital	74 children and adolescents (49% male) with CL/P aged 7-17 years	Original clinical cut-off scores	Malay version of the Strengths and Difficulties Questionnaire	INDIVIDUALS: Developmental Trajectory, Social Integration
	Hussin et al, 2022^21^	To identify cultural beliefs about the causes of CL/P among parents in a multiethnic society	Quantitative, cross-sectional, descriptive, interview based on a questionnaire	Three hospitals	295 caregivers (222 biological mothers, 62 biological fathers, 11 other relatives)	N/A	Nonvalidated questionnaire in local languages	CAREGIVERS: Social Experiences, Emotional Well-Being, Treatment Experiences
	Noor et al, 2007^43^	To determine the psychosocial status and health satisfaction in adolescents with CL/P and their parents	Quantitative, cross-sectional, questionnaire administered via interview	Single university hospital	60 adolescents with CL/P aged 12-17 years and their parents	None	Adapted Child and Parent Interview Schedules, Cleft Evaluation Profile	CAREGIVERS: Social Experiences, Treatment Experiences INDIVIDUALS: Social Integration, Emotional Health, Satisfaction with Treatment
	Norsa’adah et al, 2024^48^	To describe the level of stress and types of coping strategies used by parents of children with CL/P	Quantitative, cross-sectional, questionnaire administered via interview	1 hospital, 1 dental clinic	84 parents (71 mothers and 13 fathers) of children with CL/P younger than 12 years	N/A	Malay versions of the Depressive, Anxiety and Stress Scale-42 and COPE inventory	CAREGIVERS: Emotional Well-Being
Philippines (Lower-middle income)	Daack-Hirsch et al, 2010^32^	To describe beliefs about the cause, prevention, and treatment of CL/P	Qualitative, structured interview	4 cities	27 parents of children with CL/P, 31 parents of children without CL/P, 22 healthcare workers	N/A	N/A	CAREGIVERS: Social Experiences
	Felipe-Dimong et al, 2022^33^	To explore the causal beliefs of mothers of children diagnosed with CL/P	Qualitative, semi-structured interviews	Single tertiary hospital	2 mothers of children with CL/P (extracted from a sample of 18 mothers of children with congenital conditions)	N/A	N/A	CAREGIVERS: Social Experiences
Thailand (Upper-middle income)	Chuacharoen et al, 2009^61^	To describe the felt needs of parents of infants with CL/P	Qualitative, structured interviews	Two hospitals	15 parents (12 mothers and 3 fathers) of children with CL/P aged 0-3 months	N/A	N/A	CAREGIVERS: Treatment Experiences INDIVIDUALS: Developmental Trajectory
	Namchaitaharn et al, 2021^68^	To evaluate treatment and breastfeeding rate in infants with CL/P	Quantitative, review of medical records at 4 time points	Single university hospital	35 mother-infant dyads (57% female) aged 0-7+ days	None	N/A	INDIVIDUALS: Developmental Trajectory
	Patjanasoontorn et al, 2012^51^	To determine quality of life and ongoing care needs of children with CL/P and the mental health of their parents	Quantitative, cross-sectional, questionnaire	Single center	36 children (50% male) with CL/P aged 5 years and their parents	Original clinical cut-off scores	Nonvalidated questionnaires, Thai version of the General Health Questionnaire-12	CAREGIVERS: Emotional Well-Being, Treatment Experiences
	Pisek et al, 2014^67^	To determine oral health-related quality of life in children with CL/P	Quantitative, case–control, questionnaire	Single hospital	68 children and adolescents (50% male) with CL/P aged 10-14 years	118 children (41% male) without CL/P aged 10-14 years	Thai version of the Child-Oral Impacts on Daily Performance Index	INDIVIDUALS: Developmental Trajectory, Emotional Health, Health-Related Quality of Life
	Pongpagatip et al, 2012^34^	To determine knowledge and service satisfaction in caregivers of children with CL/P	Quantitative, cross-sectional, questionnaire	Single center	106 caregivers of children with CL/P (child age unavailable)	None	Nonvalidated questionnaire	CAREGIVERS: Social Experiences, Treatment Experiences
	Wanicharoen et al, 2023^76^	To explore health-related quality of life in children and adolescents with CL/P	Qualitative, semi-structured interviews	Single university clinic	8 children and adolescents with CL/P aged 9-14 years	N/A	N/A	INDIVIDUALS: Developmental Trajectory, Social Integration, Emotional Health
Vietnam (Lower-middle income)	van Giap et al, 2023^36^	To assess awareness of CL/P among families of children with CL/P	Quantitative, cross-sectional, descriptive, questionnaire administered via interview	Single hospital	196 families of children with repaired CL/P aged 5 months-17 years	N/A	Nonvalidated questionnaire	CAREGIVERS: Social Experiences, Emotional Well-Being, Treatment Experiences INDIVIDUALS: Developmental Trajectory, Social Integration
Europe and Central Asia								
Turkey (Upper-middle income)	Aslan et al, 2018^65^	To identify variables affecting family function and life quality in parents of children with CL/P	Quantitative, cross-sectional, questionnaire	Two clinics at single University	148 parents of 74 children with CL/P aged 0-18 years	144 parents of children without CL/P aged 0-18 years	Adapted version of the Family Assessment Scale, short form World Health Organization Quality of Life scale	CAREGIVERS: Quality of Life
	Boztepe et al, 2020^46^	To explore parenting stress and factors affecting mothers of infants with CL/P	Quantitative, case–control, questionnaire	Single university hospital	90 mothers of children with CL/P aged 0-12 months (45 pre-operative, 45 postoperative)	90 mothers of children without CL/P	Turkish adaptations of the Parenting Stress Index Short Form, Multidimensional Scale of Perceived Social Support	CAREGIVERS: Emotional Well-Being
	Çinar et al, 2020^57^	To determine why and how parents of children with CL/P use Facebook	Mixed methods data extracted from online support groups	Online Facebook groups	7695 users of 8 group accounts	N/A	N/A	CAREGIVERS: Treatment Experiences
	Erdost et al, 2023^47^	To determine parenting stress status among parents of infants with CL/P	Quantitative, cross-sectional, questionnaire	Single hospital	100 parents (60 mothers and 40 fathers) of children with CL/P, majority under 1 year or age	N/A	Parenting Stress Index Short Form in Turkish	CAREGIVERS: Emotional Well-Being
Latin America and the Caribbean								
Argentina (Upper-middle income)	Wyszynski et al, 2005^63^	To study the social environment of families of children with and without CL/P	Quantitative, cross-sectional, questionnaire	Single clinic	165 parents of children with CL/P aged 2-11 years	180 parents of children without CL/P (child age unavailable)	Moos Family Environment Scale in Spanish	INDIVIDUALS: Health-Related Quality of Life
Brazil (Upper-middle income)	Carvalho et al, 2021^44^	To evaluate the emotional and social experiences of caregivers of children with CL/P	Quantitative, cross-sectional, questionnaire administered via interview	Single hospital	41 caregivers (87% female) of children with CL/P (child age unavailable)	N/A	Nonvalidated questionnaire in Portuguese	CAREGIVERS: Emotional Well-Being, Treatment Experiences INDIVIDUALS: Developmental Trajectory
	Corrêa de Queiroz Herkrath et al, 2018^71^	To investigate the determinants of health-related and oral health -related quality of life among adults with CL/P	Quantitative, cross-sectional, questionnaire administered by interview	Single center	10 adults with CL/P aged 18+ years	None	Medical Outcomes Study 36-item Short-Form Survey, Oral Impacts on Daily Performance questionnaire, Cleft Evaluation Profile	INDIVIDUALS: Developmental Trajectory, Satisfaction with Treatment, Health-Related Quality of Life
	Cunha et al, 2021^84^	To evaluate the correlation between religiosity, spirituality, and self-esteem in adolescents with CL/P	Quantitative, cross-sectional, questionnaire	Single hospital	100 adolescents and adults with repaired CL/P aged 12-18 years	None	Translated version of the Duke University Religion Index, Rosenberg Self-Esteem Scale	INDIVIDUALS: Emotional Health
	da Silva et al, 2017^73^	Evaluate the impact of cleft on oral health-related quality of life	Quantitative, cross-sectional, questionnaire	Single center	231 individuals (103 female, 128 male) with CL/P aged 7-65 years	None	Index of Oral Impacts on Daily Performances in Portuguese	INDIVIDUALS: Developmental Trajectory, Health-Related Quality of Life
	Mariano et al, 2018^70^	To assess the prevalence and impact of orofacial dysfunction and quality of life in adults with and without CL/P	Quantitative, case–control, questionnaire	Single hospital	60 adults (50% female) with CL/P aged 31-65 years	60 adults without CL/P (50% female) aged 31-65 years	Brazilian version of the Nordic Orofacial Test–Screening and the 36-Item Short Form Survey	INDIVIDUALS: Developmental Trajectory
	Palmeiro et al, 2018^74^	Compare quality of life, pain, depression, masticatory ability and bite force in adults with and without CL/P	Quantitative, cross-sectional, questionnaire	Single hospital	20 adults with CL/P aged 20-42 years	20 maxillary denture wearers aged 53-72 years and 20 controls aged 23-24 years	Oral Health Impact Profile-14 and the Research Diagnostic Criteria	INDIVIDUALS: Developmental Trajectory, Emotional Health, Health-Related Quality of Life
	Razera et al, 2017^62^	To examine stress in caregivers of children with CL/P	Quantitative, cross-sectional, questionnaire administered via interview	Single hospital	100 caregivers (98% mothers) of children with CL/P aged 2-18 months	None	Brazilian version of the Burden Interview Scale	CAREGIVERS: Treatment Experiences
	Tannure et al, 2013^87^	To assess the quality of life of children previously treated for CL/P compared with children without CL/P	Quantitative, case–control, questionnaire	Single hospital	35 children (60% male) with CL/P aged 6-10 years	35 children without CL/P aged 6-10 years	Portuguese version of the Autoquestionnaire Qualité de Vie Enfant Imagé	INDIVIDUALS: Health-Related Quality of Life
Colombia (Upper-middle income)	González-Carrera et al, 2022^53^	To identify barriers to the comprehensive management of CL/P from the perspective of caregivers	Mixed methods, cross-sectional, questionnaire and focus group	Single center	50 parents (78% female) of children (44% female) with CL/P aged 4-10 years	None	Previously validated questionnaire	CAREGIVERS: Emotional Well-Being, Treatment Experiences INDIVIDUALS: Developmental Trajectory
El Salvador (Upper-middle income)	Aleman et al, 2021^30^	To describe coping strategies used by parents of children with CP±L during their child's early development	Qualitative, semi-structured interviews	Single center	16 parents (9 mothers, 7 fathers) of children with CP±L aged 6 months-6 years	N/A	N/A	CAREGIVERS: Social Experiences, Emotional Well-Being, Treatment Experiences INDIVIDUALS: Developmental Trajectory, Social Integration
Honduras (Upper-middle income)	Rivera et al, 2013^59^	To assess parental satisfaction with their child's cleft lip/palate surgery	Quantitative, pre- and postsurgery structured interviews	Medical mission	Mothers and fathers of 45 children (3 months-17 years) (22 children at follow-up)	N/A	N/A	CAREGIVERS: Treatment Experiences
Middle East and North Africa								
Iran (Upper-middle income)	Eslami et al, 2013^81^	To investigate oral health-related quality of life in individuals with CL/P	Quantitative, cross-sectional, questionnaire	Single dental school	50 children and adolescents (26 female) with nonsyndromic CL/P aged 8-15 years	None	Child Oral Health Impact Profile	INDIVIDUALS: Emotional Health
	Hasanpour et al, 2017^69^	To investigate feeding behavior in children with CL/P and parental responses	Quantitative, cross-sectional, questionnaire	Single university clinic	120 parents of children with CL/P aged 6 months-6 years	None	Questionnaire based on the Montreal Children's Hospital Feeding Scale and Behavioral Pediatrics Feeding Assessment Scale	INDIVIDUALS: Developmental Trajectory
	Hasanzadeh et al, 2014^50^	To investigate coping strategies, levels of psychological distress and family impact in mothers of children with CL/P	Quantitative, cross-sectional, questionnaire	1 clinic, 2 university hospitals	55 mothers of children with CL/P aged 9-16 years	Original clinical cut-off scores	Iranian versions of the Family Impact Scale, General Health Questionnaire-12, and Coping Response Inventory	CAREGIVERS: Emotional Well-Being
	Hemati et al, 2017^55^	Investigate the effect of Fordyce Happiness program on mothers of children with CL/P	Quantitative, intervention	Single university clinic	32 mothers	32 mothers of children without CL/P	Family Performance Questionnaire	CAREGIVERS: Emotional Well-Being
Jordan (Lower-middle income)	Alfwaress et al, 2020^23^	To explore social and religious attitudes of parents of children with CL/P and predictors of beliefs and behaviors	Quantitative, cross-sectional, questionnaire	Single hospital	153 parents (192 mothers, 61 fathers) of children with CL/P aged 0-20 years	None	Nonvalidated questionnaire in Arabic	CAREGIVERS: Social Experiences INDIVIDUALS: Social Integration
	El-Hneiti et al, 2024^42^	Evaluate the social and emotional concerns of mothers of children with cleft palate	Quantitative, cross-sectional, questionnaire	Single hospital	156 Jordanian mothers of children with cleft palate aged under 18 years	Control group, 156 mothers of children without cleft palate	Nonvalidated 5-point Likert questionnaire in Arabic	CAREGIVERS: Social Experiences, Emotional Well-Being
Syria (Low income)	Dak-Albab, 2013^80^	To investigate the impact of socioeconomic status on oral health-related quality of life among children with CL/P	Quantitative, cross-sectional, questionnaire	Single university clinic	87 children with CL/P (child age unavailable)	None	Arabic version of the Child Oral Health-Related Quality of Life questionnaire	INDIVIDUALS: Developmental Trajectory, Health-Related Quality of Life
South Asia								
India (Lower-middle income)	Behal et al, 2016^64^	To determine the quality of life of parents of children with CL/P in comparison to a control group	Quantitative, cross-sectional, questionnaire	Single dental school	20 parents (10 mothers, 10 fathers) of children with CL/P aged 0-3 years	20 parents (11 mothers, 9 fathers) of children without CL/P aged 0-3 years	Hindi version of the short form World Health Organization Quality of Life scale	CAREGIVERS: Quality of Life
	Gowda et al, 2013^49^	To investigate mental health and quality of life in primary caregivers of children with CL/P	Quantitative, cross-sectional, questionnaire	Single hospital	79 caregivers (81% female) of children with CL/P aged 0-12 years	None	General Health Questionnaire-12, World Health Organization Quality of Life scale in English and Kannada	CAREGIVERS: Emotional Well-Being, Quality of Life
	Karikalan et al, 2022^88^	To evaluate health-related quality of life in children with CL/P	Quantitative, cross-sectional, questionnaire	Single hospital	60 children with CL/P aged 2-6 years	None	Tamil version of the Early Childhood Oral Health Impact Scale	INDIVIDUALS: Health-Related Quality of Life
	Nagappan et al, 2019^86^	To assess oral health-related quality of life among children with CL/P	Quantitative, case–control, questionnaire	Single hospital	80 children and adolescents (63·8% male) with CL/P aged 8-16 years	80 children (55% male) without CL/P aged 8-16 years with no history of systemic disease	Child Oral Health Impact Profile	INDIVIDUALS: Health-Related Quality of Life
	Naram et al, 2013^29^	To understand cultural perspectives of CL/P	Qualitative, semi-structured interviews	Single surgical center	23 parents of children with CL/P (average age: 28 months)	N/A	N/A	CAREGIVERS: Social Experiences, Treatment Experiences
	Sivanagini et al, 2018^52^	To assess the prevalence of anxiety and depression in mothers of children with cleft palate before and after the delivery of feeding plate obturators	Quantitative, cross-sectional, questionnaire	Single dental college	30 mothers of children with CL/P aged 10 days-3 months	Original normative data	Hospital Anxiety and Depression Scale	CAREGIVERS: Emotional Well-Being
	Subramaniyan et al, 2018^60^	To document caregiver perceptions of communication status and needs in children with repaired CLP	Qualitative, 6 focus groups	2 hospitals	55 caregivers of children with CL/P aged 5-12 years	N/A	N/A	CAREGIVERS: Treatment Experiences INDIVIDUALS: Developmental Trajectory, Social Integration
	Weatherley-White et al, 2005^20^	To identify parental perceptions of causal beliefs and child social interaction	Qualitative, structured interviews	Medical mission, single hospital	52 parents of children with CL/P (child age unavailable)	N/A	N/A	CAREGIVERS: Social Experiences, Treatment Experiences INDIVIDUALS: Developmental Trajectory, Social Integration
	Wydick et al, 2022^72^	To estimate the effects of CL/P status and surgery on the physical, social, and mental well-being of children	Quantitative, case–control, questionnaire	Medical mission in 5 states	276 adolescents and adults with CL/P aged 11-19 years (238 received at least one surgery, 38 unoperated)	276 siblings/ cousins, 566 controls	Annual Statistics of Education Report survey	INDIVIDUALS: Developmental Trajectory, Social Integration, Emotional Health, Health-Related Quality of Life
Pakistan (Lower-middle income)	Shafiq et al, 2022^40^	To assess the relationship between emotional distress, social isolation, and psychological well-being among caregivers of children with CL/P	Quantitative, cross-sectional, questionnaire	Single hospital	200 caregivers (104 mothers and 96 fathers)	N/A	Urdu versions of the Caregiver Social Isolation Scale, the Depression, Anxiety, Stress Scale-21, and a Psychological Well-Being questionnaire derived from Boztepe et al, 2020	CAREGIVERS: Social Experiences, Emotional Well-Being
Sub-Saharan Africa								
Benin (Lower-middle income)	Habersaat et al, 2014^37^	To identify practical and cultural factors influencing the mental health of mothers of children with CL/P	Quantitative, cross-sectional, questionnaire administered via interview	1 Beninese hospital, 1 Swiss hospital	36 mothers of children with CL/P (mean age: 2 months)	40 Swiss mothers of children with CL/P (mean age 34 months)	Perinatal Posttraumatic Stress Questionnaire, Beck Depression Inventory in French or local language	CAREGIVERS: Social Experiences, Emotional Well-Being, Treatment Experiences
Ethiopia (Low income)	Abebe et al, 2018^66^	To assess oral health-related quality of life in children with CL/P	Quantitative, cross-sectional, questionnaire	Single hospital, single University dental clinic	41 children and adolescents (51% male) with CL/P aged 10-15 years	None	Amharic translation of the Child Oral Health Impact Profile	CAREGIVERS: Quality of Life INDIVIDUALS: Health-Related Quality of Life
Ghana (Lower-middle income)	Bonsu et al, 2018^26^	To explore the reactions and psychosocial experiences of mothers having a child with CL/P	Qualitative, semi-structured interview	Single hospital	12 mothers of children with CL/P aged 3-12 weeks	N/A	N/A	CAREGIVERS: Social Experiences, Emotional Well-Being, Treatment Experiences INDIVIDUALS: Developmental Trajectory
	Dapaah et al, 2021^39^	To explore the reaction of mothers to seeing their child with CL/P for the first time	Qualitative, semi-structured interview	Single tertiary health facility	12 mothers of children with CL/P (child age unavailable)	N/A	N/A	CAREGIVERS: Social Experiences, Emotional Well-Being
	Sommer et al, 2021^25^	To examine practices regarding CL/P among medical professionals and caregivers of children with CL/P and identify barriers and facilitators to comprehensive cleft care	Qualitative, semi-structured interviews and a focus group	Single hospital	45 caregivers of children with CL/P (child age unavailable), 1 adult with CL/P, 13 CL/P team members	N/A	N/A	CAREGIVERS: Social Experiences, Emotional Well-Being, Treatment Experiences INDIVIDUALS: Developmental Trajectory, Social Integration
	Tano et al, 2022^41^	To explore the expectations of caregivers of children with CL/P attending their first cleft clinic	Qualitative, semi-structured interviews in English or local language	Single hospital	20 caregivers (14 mothers, 4 fathers, 2 grandmothers) of infants with CL/P	N/A	N/A	CAREGIVERS: Social Experiences, Emotional Well-Being, Treatment Experiences
Kenya (Lower-middle income)	Kimotho et al, 2020^31^	To examine the experiences of parents of children with CL/P, the components of CL/P stigma, and cultural beliefs	Qualitative, semi-structured interviews	Single hospital	24 parents (22 mothers and 2 fathers) of children with CL/P (child age unavailable)	N/A	N/A	CAREGIVERS: Social Experiences, Emotional Well-Being
Nigeria (Lower-middle income)	Adeyemo et al, 2016^19^	To explore stigmatization, discrimination, and sociocultural inequalities in families of children with CL/P	Quantitative, cross-sectional, questionnaire administered via interview	Single hospital	51 mothers of children with CL/P (child age unavailable)	None	Nonvalidated questionnaire in English and local languages	CAREGIVERS: Social Experiences, Emotional Well-Being
	Fadeyibi et al, 2012^83^	To examine the psychosocial effect of CL/P	Quantitative, cross-sectional, questionnaire	Single university hospital	116 individuals (49% male) with CL/P aged 0-64 years, parent-report under the age of 12 years	Original norms and clinical cut-off scores	General Health Questionnaire-28, State-Trait Anxiety Inventory, Self-Rating Depression Scale	INDIVIDUALS: Emotional Health
	Olasoji et al, 2007^18^	To identify perceptions of etiology in mothers of children with CL/P	Qualitative, structured interviews	Two university teaching hospitals	36 mothers of children with CL/P (child age unavailable)	N/A	N/A	CAREGIVERS: Social Experiences
South Africa (Upper-middle income)	Hlongwa et al, 2018^38^	To obtain caregivers’ perceptions of health service provision and support for children with CL/P	Qualitative, semi-structured interviews	11 university hospitals	79 caregivers of children with CL/P aged 0-8 years	N/A	N/A	CAREGIVERS: Social Experiences, Emotional Well-Being, Treatment Experiences INDIVIDUALS: Developmental Trajectory, Social Integration
	Patel et al, 2003^77^	To explore the perceptions of adults with repaired CL/P regarding their quality of life	Qualitative, semi-structured interviews	Snowball sampling via healthcare professionals	20 adults (70% male) with CL/P aged 18-50 years	N/A	N/A	INDIVIDUALS: Developmental Trajectory, Social Integration, Emotional Health
Uganda (Low income)	Kesande et al, 2014^28^	To determine the prevalence, pattern, and perceptions of CL/P	Mixed methods, medical chart review and semi-structured interviews	Two hospitals	20 mothers of children with CL/P and 24 healthcare staff	N/A	N/A	CAREGIVERS: Social Experiences, Emotional Well-Being INDIVIDUALS: Developmental Trajectory
	Luyten et al, 2013^58^	To assess parental satisfaction with speech and facial appearance following synchronous lip and palate closure	Quantitative, cross-sectional, questionnaire	Single hospital	44 caregivers (26 mothers, 13 fathers, 5 guardians) of children with CL/P with a mean age 3·1 years	Control group, 44 foster mothers of children with a mean age of 3·7 years	Cleft Evaluation Profile	CAREGIVERS: Treatment Experiences
	Nabatanzi et al, 2021^35^	To explore maternal perceptions, experiences with breastfeeding, and support received among mothers of children with CL/P	Mixed methods, questionnaire, focus groups, semi-structured interviews	Single hospital	32 mothers of children with CL/P aged 0-24 months	N/A	Adapted Feeding Practices questionnaire in Luganda	CAREGIVERS: Social Experiences, Emotional Well-Being, Treatment Experiences INDIVIDUALS: Developmental Trajectory
Zimbabwe (Lower middle income)	Mzezewa et al, 2010^24^	To determine the feelings of parents and the wider public towards infants with CL/P and establish perceived causes and postpartum marital status	Quantitative, cross-sectional, questionnaire administered via interview	Single hospital	124 parents (120 mothers and 4 fathers) of children with CL/P aged 4 days-2 years	None	Nonvalidated questionnaire	CAREGIVERS: Social Experiences
Multiple Geographical Regions								
China, Colombia, Thailand (All upper-middle income)	Black et al, 2009^45^	To compare maternal reactions toward the birth of a child with CL/P across cultures	Quantitative, case–control, questionnaire	Two Thai hospitals, single hospitals in China and Colombia	197 mothers (98 mothers of children with CL/P and a historical cohort of 99 US mothers of children with CL/P)	None	Adapted version of the When My Child Was Born questionnaire translated into Thai, Chinese, Spanish, Uygur	CAREGIVERS: Emotional Well-Being
Cambodia, Egypt, Kenya, India, Peru (lower-middle income/upper-middle income)	Mednick et al, 2013^27^	To describe and compare causal beliefs associated with CL/P across countries	Qualitative, single question extracted from structured interviews	Medical mission clinics	279 parents of children with CL/P and adults with CL/P aged 0-75 years	N/A	N/A	CAREGIVERS: Social Experiences

Abbreviation: CL/P, cleft lip and/or palate.

^a^ Based on World Bank Income Classification (2024).

## Caregivers of Children With CL/P

The articles included in this review examined a range of psychosocial domains relating to caregivers of children and adolescents with CL/P. These included: Social Experiences, Emotional Well-Being, Treatment Experiences, and overall Quality of Life (QoL).

### Social Experiences

Twenty-five studies reported on caregivers’ social experiences. Subthemes included causal attributions (17 articles) and societal stigma (15 articles).

#### Causal attributions

Caregivers of children born with CL/P often attributed the cause of their child's cleft to nonmedical explanations. Across two Nigerian studies, a large proportion of caregivers believed their child's CL/P was an “act of God.”^18,19^ Others perceived CL/P to be caused by “wicked people,” “ancestral punishment,” or an “evil spirit.”^18,19^ Similarly, caregivers of children with CL/P commonly attributed CL/P to “God's will” in India,^20^ Malaysia,^21^ and Indonesia,^22^ to a “punishment from God” in Jordan,^23^ to “witchcraft” in Zimbabwe^24^ and Ghana,^25^ and to a “curse” in Ghana.^26^ “Supernatural” causes were frequently reported by caregivers in Kenya, India, Egypt,^27^ Malaysia,^21^ and Uganda^28^ and were also associated with cleft type in one study which utilized a nonvalidated questionnaire (cleft lip; Jordan).^23^ In India, more than half of the 23 mothers interviewed believed CL/P was caused by an eclipse,^29^ which was also identified as a likely cause by mothers in El Salvador (n = 16).^30^ Interviews with 24 caregivers in Kenya pointed to other natural phenomena, such as lightning or earthquakes.^31^ Other common external attributions included the use of the contraceptive pill, the use of unprescribed medication, infidelity (Nigeria, Indonesia),^20,22^ and accidents while pregnant (the Philippines, Malaysia).^21,32,33^ Caregivers also reported internal attributions (Peru, Egypt, Jordan, India, Kenya, and El Salvador^20,23,27,30,31^), such as the mother's diet, medication use, stress, past sins, an assumption they were carrying a sexually transmitted disease, or other behaviors (such as pregnant mothers not taking appropriate care of themselves or not attending hospital appointments during the pregnancy), as well as observing or reprimanding a person with CL/P during the first 3 months of pregnancy (the Philippines).^32^ In four studies, few caregivers acknowledged the role of genetics, despite a positive family history (India, Nigeria, Malaysia, Indonesia).^19–22^ In three other studies, caregivers believed CL/P could be an inherited condition (Kenya, Zimbabwe, and the Philippines).^24,31,32^ In Thailand, caregivers demonstrated a moderate level of knowledge of the etiology of CL/P.^34^ Across several other countries, many caregivers reported not knowing or not understanding the cause of CL/P (Nigeria, Malaysia, Indonesia, Kenya, Cambodia, Egypt, Peru, India).^19,21,22,27^

#### Societal stigma

Cultural beliefs about the etiology and implications of CL/P could lead to or exacerbate the stigma felt by caregivers. Participants in Nigeria and Kenya reported being avoided by neighbors, relatives, and friends because of their child's cleft, with people also refusing to touch the child for fear of something bad happening to them.^19,26,29,31^ Fifty-six percent of 32 mothers participating in a Ugandan study reported persistent social stigma using a nonvalidated questionnaire.^35^ While caregivers in Zimbabwe^24^ and Vietnam^36^ felt the public was generally sympathetic toward the family, some also described instances of their child being laughed at being or regarded as an “evil creature.” Caregivers in Kenya, South Africa, Ghana, and Benin also reported their child being stared at or talked about unfavorably.^25,31,37,38^ Mothers experienced difficult interactions with members of their husband's family (Nigeria, El Salvador, and Ghana)^19,25,26,30^ with some mothers reporting that their husband felt ashamed of their child.^19^ In Zimbabwe, 19% of 124 participating mothers were divorced, and 23% had been abandoned by their husband as a result of having a child with CL/P.^24^ Fathers in Kenya, El Salvador, South Africa, and Ghana had also rejected their child and some had ultimately left the family home, according to 4 qualitative studies.^26,30,31,38^ In contrast, some studies described unconditional support provided by the father.^39^ Social isolation and exclusion, either enacted by members of the community or self-imposed, were recounted in caregiver reports in eight countries (Nigeria, Kenya, Benin, South Africa, Vietnam, Pakistan, Jordan, and Ghana).^19,25,26,31,36–38,40–42^ Conversely, two studies highlighted the complexities of seeking treatment for CL/P due to societal beliefs about Western medicine. Namely, some religious groups believe pursuing cleft surgery to be “against God's will” (Benin, Nigeria).^20,39^ As a result, some groups of caregivers had consulted traditional healers about their child's CL/P.^18^

### Emotional Well-Being

Twenty-eight studies reported on caregivers’ emotional well-being. Subthemes included caregivers’ emotional reactions to the birth of a child with CL/P (11 articles), the emotional impact of having a child with CL/P (14 articles), and caregivers’ coping strategies (8 articles).

#### Emotional Reactions

Caregivers’ knowledge of CL/P prior to their baby's diagnosis/birth varied considerably. In response to their child's diagnosis/birth, caregivers reported a range of conflicting emotional reactions. This included shock or surprise, fear, disappointment, worry, sadness, happiness, hopelessness, guilt, disbelief, denial, and shame.^26,28,30,31,36,38,39,41,43,44^ Some caregivers qualitatively described the birth of a baby with CL/P to be a traumatic experience^30^ (n = 16; El Salvador), which was reported to impact parent–infant bonding in two additional qualitative studies (Ghana).^26,39^ Largely in response to societal stigma, 73% of 51 Nigerian mothers felt ashamed of their child and 26% wished their child had not been born.^19^ Some mothers in Nigeria, Kenya, and Ghana disclosed wanting to abandon their baby and/or that they had considered infanticide.^19,31,39^ One study identified potential differences in maternal affect scores across countries (Thailand, China, Colombia) using an adapted measure, with Chinese mothers reporting the least positive feelings about their infant.^45^

#### Emotional Impact

Depressive symptoms, anxiety, and stress were qualitatively described by mothers in Kenya (n = 24), with some participants having contemplated suicide.^31^ In Turkey, mothers of children with CL/P reported significantly higher parenting stress and lower levels of social support during the child's first year of life than a control group,^46^ and fathers reported greater parenting stress than mothers.^47^ A notable proportion of Malaysian caregivers also reported mild to severe stress.^48^ In India, more than half of participating parents reported poor mental health.^49^ Seventy-six percent of 32 mothers in Uganda reported anxiety on a nonvalidated measure.^35^ Mothers of children with CL/P in Jordan reported greater anxiety, depressive symptoms, worry for the future, and isolation than controls, also according to a nonvalidated measure.^42^ Depressive symptoms were found to be higher in Beninese mothers living in urban areas (n = 36).^37^ Poor emotional well-being was still evident in caregivers of older children (aged 9-15 years), with 38.2% of 55 mothers in Iran reporting psychological distress, and 23.6% suspected of having “severe” psychological difficulties.^50^ However, Patjanasoontorn did not identify mental health difficulties in^36^ Thai caregivers using the same outcome measure.^51^ Beninese mothers of children with CL/P (n = 36) were at higher risk of depression and posttraumatic stress symptoms compared to mothers of children with CL/P in Switzerland.^37^ No difference in depression and anxiety scores was found between mothers in India and German population norms (n = 30).^52^ In two qualitative studies, mothers described an ongoing emotional burden of caring for a child with CL/P (Kenya and South Africa).^31,38^ Family well-being was negatively impacted by a lack of social support,^40,42,46^ feeding difficulties, surgical experiences,^46^ lower household income, and lower education level.^21^

#### Coping Strategies

Caregivers drew upon a range of strategies to help them cope with the challenges of having a child with CL/P. This included seeking support (Nigeria, Malaysia, and Iran),^19,48,50^ making positive appraisals (Iran),^50^ and problem-solving (Iran and Malaysia).^48,50^ Some caregivers described receiving emotional, practical, and financial support from close family members and members of their church (Kenya, South Africa, Brazil, Uganda, and Ghana).^26,31,35,38,39,44^ Caregivers in Colombia and Ghana emphasized the need for local peer support groups.^25,26,53^ In a qualitative study in El Salvador, Aleman and colleagues observed caregivers to use emotion-focused strategies initially, and problem-focused strategies later on, the latter of which led them in search of treatment (n = 16).^30^ In Iran and Malaysia, mothers of children with CL/P exhibited greater psychological distress if they relied upon avoidant-oriented coping strategies.^48,50^ In one Chinese study, hope, perceived social support, optimism, and higher coping scores predicted greater resilience in mothers and fathers, while lower resiliency was negatively associated with higher levels of parenting stress.^54^ Insecure job status and higher medical costs were also associated with lower resiliency among fathers.^54^ In an intervention study based on the Fordyce Happiness program (n = 32), Hemati and colleagues (Iran) found significant improvements in mothers’ well-being and coping skills after participation in the program compared to a control group.^55^

### Treatment Experiences

Twenty-four studies reported on caregivers’ treatment experiences as a factor influencing caregiver psychosocial well-being. Subthemes included caregivers’ satisfaction with healthcare (23 articles), and the perceived burden of care (9 articles).

#### Satisfaction with Health Care

Reports of antenatal detection rates varied greatly, which affected the timing of the diagnosis and negatively impacted caregivers’ subsequent healthcare experiences. In one Brazilian study of 41 caregivers, only 9.8% of caregivers had received their child's diagnosis of CL/P prenatally and 48.8% reported having never received an adequate explanation of their child's condition.^44^ Following the birth, some caregivers felt they had not been treated kindly by noncleft specialist healthcare providers and recounted their baby quickly being taken away (South Africa and Benin).^37,38^ Caregivers perceived noncleft specialists to lack awareness of CL/P and consequently received inaccurate guidance or a lack of referral to an appropriate cleft center (South Africa, Malaysia, El Salvador, Banten, and Ghana).^21,25,26,30,38,56^ Most families were unfamiliar with the treatment process or where to seek help (Ghana, Colombia, Vietnam, Indonesia)^22,36,41,53^ and often struggled to access adequate information (South Africa, El Salvador, Benin, Indonesia, Thailand, Ghana, Vietnam).^30,36–39,51,56^ This lack of awareness among parents and noncleft specialists could lead to delayed treatment in some cases, with only 18.9% of children from 196 families receiving any treatment before 6 months of age in one study (Vietnam).^36^ Some caregivers had sought information from online support groups (Turkey and Vietnam),^36,57^ particularly in relation to surgery, feeding, and financial and spiritual support.^57^ In contrast, satisfaction with the care provided by specialist multidisciplinary teams was high according to three small studies (India, Malaysia, and Colombia).^29,41,53^ In Thailand, caregivers reported being content with their ability to access the local cleft center, the information they received regarding CL/P and its treatment, and available health insurance, as well as the support they received from members of the team, using a nonvalidated questionnaire.^34^ Mothers in South Africa and Ghana had been offered information, counselled, and reassured their child would receive high-quality treatment, according to three qualitative studies.^26,38,39^ Most Malaysian parents felt “at ease” attending the clinic, felt able to share their concerns with clinicians, felt involved in treatment decisions, and felt treatment information was easy to understand.^43^ Caregivers qualitatively reported being happy with their child's primary surgical treatment (South Africa, El Salvador, India, Malaysia),^20,30,38,43^ including postsurgical improvements in their child's facial appearance, (Thailand, Ghana, Uganda, Brazil),^25,51,58,59^ feeding, and speech (Ghana),^25^ as identified by four small-scale studies. However, caregivers remained concerned about their child's speech in two small, quantitative studies (Uganda, Laos)^58,59^ and a minority stated the surgery had not met their expectations (Brazil, Ghana, Malaysia).^41,43,59^ Caregivers also expressed dissatisfaction with healthcare waiting times (India, Malaysia, and Colombia)^20,21,53^ and the lack of access to specialist feeding support (Uganda, Vietnam),^35,36^ speech and language therapy (India, Ghana),^25,60^ and psychosocial support (Uganda).^35^ Caregivers also requested more information about various aspects of the treatment pathway in 3 small-scale studies (Thailand, Malaysia).^43,51,61^

#### Burden of Care

Many caregivers described the burden of caring for a child with treatment needs. One Brazilian study identified moderate to severe levels of burden among 100 caregivers of children with CL/P aged 2 years and under.^62^ Hospital visits were perceived as particularly challenging, especially if travel over long distances was required to reach specialized treatment facilities (South Africa, Ghana, Honduras, Malaysia, and Colombia).^21,25,38,53,59^ Mothers qualitatively reported having to take time off work or needing to quit working altogether to meet these demands, which had a significant financial impact on the family (South Africa, El Salvador, and Ghana).^26,30,38^ In some cases, economic difficulties meant the child did not receive all available treatment (El Salvador, Malaysia, and Colombia).^21,30,53^ Unstable working conditions, a lack of health insurance, and other economic barriers to accessing comprehensive care were identified in 4 small-scale studies in Colombia, Ghana, and Thailand.^25,51,53,61^

### Quality of Life

Five studies reported on caregivers’ overall QoL. In an Argentinian study investigating family functioning, Wyszynski and colleagues found families of children with CL/P to report better family relationships, a higher level of independence, and better structure and organization than families in the control group.^63^ However, families of children with CL/P also reported participating in fewer recreational activities.^63^ Two small studies conducted in India identified lower QoL scores in caregivers of children with CL/P compared to families without CL/P.^49,64^ This was particularly true for those living in rural areas.^64^ Similar findings were reported by Aslan and colleagues, with caregivers in Turkey demonstrating poorer QoL compared to controls in relation to the physical, psychological, and social domains.^65^ However, caregivers reported good QoL overall, particularly those with older children (aged 13-18 years).^65^ In Ethiopia, Abebe et al found no differences in oral health-related QoL between 41 caregivers of children with CL/P and normative data.^66^

## Individuals Born With CL/P

The articles included in this review examined a range of psychosocial domains in relation to individuals born with CL/P. These included: Developmental Trajectory, Social Integration, Emotional Health, Satisfaction with Treatment, and Health-Related QoL.

### Developmental Trajectory

Twenty-six studies reported on the individual's developmental trajectory. Subthemes included feeding and nutrition (11 articles), physical health and function (13 articles), behavioral difficulties (3 articles), and education (10 articles).

#### Feeding and Nutrition

Feeding difficulties, aspiration, and adequate nutrition were of primary concern in relation to child development in 6 countries (South Africa, Brazil, Thailand, Ghana, Uganda, Vietnam).^25,26,35,36,38,44,61,67^ Where breastfeeding had been unsuccessful, caregivers qualitatively reported trying infant formula at great expense, as well as goat's milk (Ghana).^26^ Mothers who were given access to specialist bottles reported improved feeding in one study (Uganda).^35^ A breastfeeding promotion intervention with 35 mothers in Thailand was found to increase breastfeeding rates and reduce the length of hospital stay.^68^ Ongoing challenges in eating behaviors were observed in older children and adults in Iran, Vietnam, and Brazil.^36,69,70^

#### Physical Health and Function

Two small studies identified children with CL/P to experience recurring illness (Ghana and Uganda).^26,28^ Poor overall physical health and a low weight-to-height ratio was also highlighted (Vietnam, India, Brazil).^36,71,72^ Functional impairments were noted, such as breathing, speech, and hearing difficulties (Brazil, Thailand, India, Vietnam)^36,67,70–73^ and were found to be higher in CL/P compared to controls in two small-scale studies (Brazil and Thailand).^67,74^ In one Malaysian study, 25% of 74 children with CL/P had mild to moderate hearing loss, and a further 10.8% had persistent middle ear effusion.^75^ Focus groups carried out with caregivers in India identified a high level of concern about their children's communication abilities, with some expressing specific worries about speech errors and nasality.^60^ Caregivers were also concerned about the potential impacts of communication difficulties on their child's social and educational opportunities (India, Malaysia).^60,75^ Children and adolescents with CL/P self-reported speech difficulties in one qualitative study and indicated these difficulties could impact their ability to effectively communicate with others (n = 8; Thailand).^76^ However, one qualitative study (n = 20) found adults with CL/P to be generally satisfied with their communication abilities and speech intelligibility (South Africa).^77^ Primary CL/P surgery performed before the age of 5 years (India)^72^ and integrated speech therapy (South Africa)^77^ were reported to improve speech quality.

#### Behavioral Difficulties

Ha and colleagues (China)^78^ identified elevated parent-reported behavioral problems in a sample of 93 children with CL/P aged 6 to 11 years. Both boys and girls were reported to have more attention problems, aggressive behaviors, and withdrawn behaviors compared to controls.^78^ Boys also scored higher than controls in relation to delinquent behaviors, while destructive behaviors were more common in girls with CL/P than controls.^78^ In contrast, 98% to 100% of 104 Indonesian children with CL/P aged 6 to 15 years fell within the average range using the same measure, with only a minority of children in the sample exhibiting significant behavioral concerns according to parent-report.^79^ Goh and colleagues identified 16% to 18% of their sample of 74 children aged 7 to 17 years to be experiencing behavioral difficulties (Malaysia).^75^

#### Education

A qualitative study by Aleman and colleagues (El Salvador)^30^ found that only a minority of school-aged children in their sample were attending school, while another study identified 10% of 52 parents had actively chosen to hold their child back from attending school (India).^20^ Some caregivers stated their main reason was a fear of bullying, discrimination, or physical violence, while others felt their children would be more able to cope with school when they were older (El Salvador).^30^ One study suggested that school absences may threaten children's academic goals (Colombia),^53^ and another indicated school absences were most common among children with lower socioeconomic status (Syria).^80^ Three studies identified CL/P to have an impact on children's academic and cognitive performance (India, Thailand, China).^72,76,78^ Ha and colleagues found the highest impact on academic competency among children with more severely affected speech and/or facial appearance (China).^78^ In contrast, one study found 92.3% of 104 children to fall within the normal range in relation to school competency (Indonesia).^79^ In one small quantitative study, adults with CL/P reported at least 10 years of schooling on average (Brazil)^71^ and another sample of 20 adults qualitatively described being generally satisfied with their educational attainment and employment opportunities (South Africa).^77^ One study suggested that timely CL/P surgery could positively impact school participation (India).^72^

### Social Integration

Fourteen studies reported on social integration. Subthemes included social acceptance (10 articles) and social competence (6 articles).

#### Social acceptance

Three studies identified an occurrence of teasing, bullying, and social exclusion as reported by children, adolescents, and adults with CL/P (Malaysia, India, South Africa).^43,72,77^ In Malaysia, 75% of 60 children with CL/P aged 12 to 17 years stated they had been teased because of their cleft.^43^ Specific cleft-related features were identified as the focus of the teasing in two studies, including speech (Malaysia, India),^43,72^ and the appearance of the lips, teeth, and nose.^43^ The earliest self-reported occurrence of teasing in Malaysia was age 4 years, with the highest frequency of teasing reported at age 7 years, and a minority of children still experiencing teasing in late adolescence.^43^ One study proposed that surgery may decrease bullying and social exclusion (India),^72^ yet in another study of 60 adolescents, 67% reported still being teased after undergoing surgical and/or orthodontic treatment (Malaysia).^43^ Instances of children being teased, bullied, or rejected by peers were also reported by caregivers in Ghana,^25^ India,^20,60^ Malaysia,^43^ Jordan,^23^ and South Africa.^38^ Caregivers in India^20^ and South Africa^38^ were also concerned about their child's future marriage prospects, particularly if their child was female. In contrast, two small qualitative studies found individuals with CL/P to feel well supported by family and friends and did not believe dating had been adversely affected (Thailand, South Africa).^76,77^ In Vietnam, only 2% of 196 parents had observed their children receiving hostility or rejection by others.^36^

#### Social Competence

In India, 26% of 52 participating families exercised constraints over their child's social interaction, with a further 10% choosing to keep their child completely isolated from society.^20^ Concurrently, Soedjana and colleagues highlighted an impact of CL/P on social activities in 78.8% of their sample of 104 children (Indonesia).^79^ One study which used a nonvalidated measure found that caregivers who were less educated were more likely to keep their child hidden (Jordan).^23^ Children with CL/P in China produced lower scores of social competencies compared to children without CL/P. Children in Malaysia exhibited few prosocial behaviors.^75^ Goh and colleagues also categorized 39.2% of their sample of 74 children and adolescents as experiencing problems with peers.^75^ In contrast, 93.3% of children had average scores in relation to social competency in Indonesia,^79^ no differences were found in social activities between children with and without CL/P in China,^78^ and caregivers in Jordan denied any negativity regarding their child's ability to make friends.^23^ Some parents qualitatively reported that teaching their child how to explain CL/P to their peers had been helpful (El Salvador).^30^

### Emotional Health

Eleven studies reported on individuals’ emotional health. A higher presence of emotional difficulties compared to controls was identified in children and adults with CL/P in two small, quantitative studies (Brazil and Thailand).^67,74^ Two additional studies noted that these difficulties were more pronounced among girls (China, Iran).^81,82^ In El Salvador, parent- and self-reports identified a clinical level of concern among individuals with CL/P in relation to symptoms of depression (44.8%), state anxiety (33.6%), and trait anxiety (27.6%), as well as overall mental health (26.7%) when using US cutoff scores.^83^ Palmeiro and colleagues (Brazil) also found more symptoms of depression compared to a control group in a sample of 2074. Yet, psychological health was not found to be significantly impacted by CL/P according to Wydick and colleagues (India).^72^ While one study found overall self-esteem to be satisfactory among adolescents with CL/P (Brazil),^84^ self-confidence was found to be impacted in 83% of 60 adolescents in another (Malaysia).^43^ One qualitative study identified that speech difficulties affected individuals’ ability to communicate with others, which could in turn impact their self-confidence (n = 8; Thailand).^76^ Individuals with CL/P also discussed the importance of nurturing self-esteem in one small qualitative study (Thailand)^76^ and in another, recognizing positive outcomes, such as an increase in courage and empathy for others was important for psychosocial well-being (South Africa).^77^ No effect was found for religiosity or spirituality on adolescent self-esteem (Brazil).^84^

### Satisfaction with Treatment

Three studies reported on individuals’ satisfaction with treatment. Individuals with CL/P generally reported a high level of satisfaction with the care they had received from a specialist cleft team according to two small-scale studies (Malaysia, Brazil).^43,71^ However, 23% of 60 adolescents in a Malaysian study felt nervous attending the clinic and 22% found it difficult to discuss concerns with the clinicians.^43^ Approximately 22% of adolescents in the same study felt treatment information was difficult to understand and a minority felt they were not involved in treatment decisions.^43^ While 23% of 60 adolescents were “very satisfied” with the outcome of treatment, more than half reported that surgery had made no difference (12%) or a slight difference (52%) to their facial appearance.^43^ Prathanee and colleagues (Laos, n = 18) used a nonvalidated measure to capture that participants were least satisfied with speech articulation and the appearance of the lip.^85^ One Brazilian study highlighted a wide range of treatment outcomes among adults with CL/P (n = 10), including a significant proportion who had not undergone palatoplasty or had wide palatal fistulas.^71^ One third had never been assessed by a speech and language therapist, 80% had not undergone the recommended bone graft, and 59% had not received any orthodontic treatment.^71^ Almost 80% of the sample (n = 10) desired further treatment, most often relating to speech, nose, and teeth.^71^

### Health-Related QoL

Eleven studies reported on health-related QoL (HRQoL) in individuals with CL/P. Four studies identified significantly poorer HRQoL in individuals with CL/P compared to control groups (India, Brazil, Thailand).^67,72,74,86^ In Brazil, da Silva and colleagues found CL/P negatively impacted 27% of 231 individuals on a daily basis.^73^ Children in China rated their HRQoL as “moderate.”^82^ However, a further two studies found HRQoL in children and adolescents with CL/P to be similar to controls and/or to international norms, albeit within small samples (Ethiopia, Brazil).^66,87^ Factors found to significantly decrease HRQoL in individuals with CL/P included lower family income (Brazil, Syria),^71,80^ poor oral health (India, Brazil),^67,71,86^ use of orthodontic appliances (India),^67^ being female (Brazil),^71,73^ surgical status (ie, awaiting surgery; India),^72^ low social support (Brazil),^71^ and appearance concerns (India).^67^ Three studies identified differences in HRQoL according to cleft type (China, Brazil; n = 10-120),^71,82,87^ while one other did not (India).^88^

## Discussion

### Data Synthesis

This narrative review synthesizes the findings of 71 studies published on the psychosocial impact of CL/P in LMICs between September 2003 and July 2024. For caregivers of children with CL/P, four overarching themes were identified: Social Experiences, Emotional Well-Being, Treatment Experiences, and overall QoL. Caregiver understanding of the etiology of CL/P varied greatly, with most attributing the cause of their child's CL/P to nonmedical explanations. This subject has been studied and reviewed extensively, likely due to the impact that public perceptions can have on CL/P associated risk factors, diagnosis, and treatment.^12–14,89^ In line with previous reports,^14,16^ the current review also highlights widespread stigma and discrimination, often with significant consequences for both the family and the child. Situated in this social context, caregivers’ initial reactions to the birth of a child with CL/P were wide-ranging, with the potential to negatively impact parent–infant bonding and familial relationships. Emotional distress was found to be high in several, but not all studies, with caregivers describing a range of emotion- and problem-focused coping strategies. Early healthcare experiences were highly variable and often dependent upon the quality of information received, timely referral to specialist providers, and the perceived burden of care. Studies investigating the impact of having a child with CL/P on caregivers’ overall QoL were unable to examine variations within or between countries due to sample size.

For individuals (children, adolescents, adults) with CL/P, five overarching themes were identified: Developmental Trajectory, Social Integration, Emotional Health, Satisfaction with Treatment, and Health-Related QoL. Feeding was highlighted as a significant initial concern, followed by other cleft-related symptomology impacting physical health and function. Some behavioral difficulties were observed but require further investigation. School absences and their potential impact on educational and vocational opportunities were highlighted in some countries. The influence of societal stigma on social acceptance was evident, but findings were mixed as to whether this impacted social competency and friendships. A higher presence of emotional difficulties was identified in most of the studies reporting on this aspect of psychological adjustment, although variations in the impact on individuals’ overall health-related QoL were unable to be comprehensively examined. A generally high level of satisfaction with healthcare was reported, yet treatment outcomes were variable and evidence of dissatisfaction with aspects of care delivery was identified.

A range of risk and protective factors for psychological distress were identified by the studies included in the current review (Table 2). Such findings are helpful in facilitating the early identification of individuals and families who may be at risk. Notably, more attention to date has been paid to risk factors than to protective factors.

**Table 2.** Predictors of Psychological Distress in Individuals With CL/P and Their Caregivers.

Risk factors for psychological distress	Protective factors for psychological distress
Caregivers	
No prior knowledge of CL/P Sociocultural explanations for CL/P Societal stigma Difficult interactions with health professionals A lack of referral to a specialist centre A lack of reliable information Feeding difficulties Lengthy healthcare waiting times Low household income Insecure job status Low education level Living in a rural area Medical costs Dissatisfaction with treatment outcomes Use of avoidant coping strategies A lack of social support	Positive relationships with health professionals Specialist feeding support Accessible treatment information Involvement in treatment decisions Satisfaction with healthcare Access to social support Hope Optimism Use of problem-focused coping strategies
Individuals	
Hearing difficulties Speech difficulties School absences Teasing/bullying Social isolation/exclusion A lack of social support Gender differences Low household income Poor oral health Treatment anxiety An unoperated cleft lip or palate Poor surgical outcomes Dissatisfaction with treatment outcomes	Timely surgery Access to speech therapy Knowing how to respond to comments and questions Nurturing self-esteem

Abbreviation: CL/P, cleft lip and/or palate.

### Implications for Clinical Practice

The findings of this review call for ongoing monitoring of psychological health in individuals with CL/P and their caregivers and imply care cannot be comprehensive without taking these holistic needs into consideration. Ultimately, coordinated investment into evidence-based, multifaceted psychosocial program is needed.^90,91^ Yet, substantial challenges for the widespread implementation of comprehensive cleft care persist, including the complexity and multidisciplinary nature of the recommended approach, and a range of structural, financial, and geographical barriers. While striving to justify the resources necessary to enable access to psychosocial specialists, engagement with other strategies, such as those suggested in Table 3, may be beneficial.

**Table 3.** Recommendations for Clinical Practice and Future Research.

Recommendations for clinical practice
Consideration of need according to local context
Ongoing monitoring of psychological health in individuals and caregivers
Inform communities about scientific models of CL/P etiology and increase awareness of treatment avenues
Improvement of early detection and timely referral to a specialist provider
Improved quality and accessibility of information available to individuals and families
Engagement with schools to meet educational and social needs in the school setting
Guidance for children and caregivers on how to navigate questions, comments, staring, and teasing
Facilitation of peer support groups
Developmentally appropriate and culturally sensitive involvement of individuals and caregivers in healthcare conversations and decisions
An increase in cleft providers’ awareness of common psychosocial concerns and low-level prevention and intervention strategies
Use of neutral language when communicating with individuals and families
Recommendations for future research
Investment in high-quality local and international partnerships and research capacity-building
Research into priority areas: family functioning and healthcare satisfaction
Research into the self-reported experiences and support needs of children, adolescents, and adults with CL/P using developmentally relevant age ranges
Feasibility and acceptability testing of potential interventions as a future step
Inclusion of individuals with syndromic CL/P and related conditions
Investment in qualitative methods as complementary research tools
Consensus regarding outcome measures and collection of country/region-specific normative data to enable relevant interpretation of scores
Coordinated efforts to collect data across multiple sites
Expansion to LMICs not currently represented in international literature

Abbreviations: CL/P, cleft lip and/or palate; LMIC, low- and middle-income countries.

First, there is a need for ongoing efforts to inform communities about scientific models of CL/P etiology and increase awareness of treatment avenues.^14^ Although sociocultural explanations of illness remain prevalent in LMICs, a variety of communication methods have been found to be effective in other areas of healthcare. For example, stakeholders developed a taxonomy of interventions to increase uptake of childhood vaccinations in LMICs, which included mobile phone alerts, media and news reports, meetings with leaders to garner support, celebrity involvement in campaigns, and trained mothers visiting their peers, among others.^92^ Consistent messages delivered by trusted sources in a culturally sensitive manner could go some way to reducing the stigma felt by individuals with CL/P and their caregivers. For families with a child with CL/P, improving early detection and timely referral to specialist providers could limit the risk of misinformation, positively impact caregiver appraisals of CL/P and its treatment, and reduce the impact of misconceptions on familial and marital relationships. Early referral could also reduce unnecessary delays to treatment and increase the likelihood of optimal surgical outcomes and healthcare satisfaction. Frontline care workers should be trained on how to explain CL/P etiology to families and direct them to appropriate treatment centers.^89^ Particularly in low-resource settings, a “train the trainer” approach, whereby individual health/community workers are equipped with locally tailored skills and knowledge to deliver training to others, can be a cost-effective method of upskilling frontline staff.^93^ Improving the quality and accessibility of the information available to families and individuals with CL/P is a priority for cleft care around the world, and particularly in LMICs. Providing clear information about CL/P and its treatment,^94^ alongside psychoeducation^95^ can be a minimal but impactful intervention to promote psychological well-being. To ensure relevancy and accessibility to families and individuals, content should be codeveloped with stakeholders and different formats considered.

Encouraging school attendance for children with CL/P in LMICs and ensuring educational and psychosocial needs are met in school settings is another important step.^91^ Not only does school attendance increase educational opportunities and activities but also reduces social isolation.^96^ Children with disabilities may be held back from school due to medical appointments, and experienced or feared bullying and discrimination.^96^ Healthcare and community workers can engage with local schools to support children with CL/P entering the educational environment and to dispel myths about CL/P among teaching staff and peers.^89,97^ Caregivers and children may also benefit from guidance on how to navigate questions, comments, staring, and teasing from others.^98,99^

Cleft providers can also consider their capacity to facilitate access to peer support.^89^ In HICs, peer support groups and events are run by both CL/P teams and charities, often in collaboration, and can be held in person or online. Access to peer support provides caregivers and individuals with an opportunity to share their experiences with others, feel reassured and less alone, and more able to cope with cleft-related challenges.^100^ Recommendations on how to develop peer support groups in LMICs were recently set by an interdisciplinary stakeholder solutions group.^101^

Another clinical implication raised by this review relates to healthcare satisfaction. Broadly, healthcare satisfaction among individuals with health conditions and their caregivers tends to be high, yet examining specific aspects of healthcare in more detail identifies areas for improvement.^102^ Within this review, treatment anxiety and adherence, treatment decision-making, and burden of care were highlighted. Involving caregivers and individuals with CL/P in their care in a manner that is developmentally appropriate and culturally sensitive can ensure that families’ needs are recognized, increase communication between families and health professionals, reduce the burden of care, help children to develop problem-solving skills and resiliency, and increase well-being and satisfaction with treatment outcomes.^103–105^ Shared decision-making guidelines place an emphasis on health professionals adopting a consulting style that is curious, supportive, nonjudgmental, and unbiased.^106^ The use of evidence-based decision aids can also help to reduce health inequities,^107^ which is especially applicable to healthcare systems that are not universally accessible.^108^ How to best apply these principles in various LMIC settings is a focus for further examination.

Finally, CL/P providers from all disciplines can consider the role they play in facilitating psychosocial adjustment. Providers can increase their own awareness of common psychosocial concerns and engage with low-level prevention and intervention strategies. This could include participation in specific training workshops aimed at upskilling health professionals in fundamental psychosocial principles,^89,109^ and/or collaborative working groups designed to achieve consensus on priority areas, set out recommendations for psychosocial care, and seek solutions for overcoming barriers to implementation.^110^ Providers can also implement ways of communicating with individuals and caregivers that do not perpetuate stigma. For example, avoiding terms that hold negative connotations, such as “defect,” “abnormality,” and “disfigurement” and instead using neutral terms, such as “diagnosis” or “cleft lip and palate” can make an important difference to the way families and individuals perceive the condition.^111^ Similarly, using person first language (“child with a cleft” instead of “cleft child”) indicates that the child is seen as a whole person, while saying “cleft surgery” rather than “cleft repair” remains accurate without unintentionally implying the child is somehow “broken.”^111^

### Implications for Future Research

Academic research emphasizing the relevance and significance of psychosocial care in LMICs is an essential tool for onboarding management and advocating with funders.^110^ Investing in high-quality research would also ensure that psychosocial program are evidence-based, safe, cost-effective, and as impactful for the community as possible. Building practice-relevant research capacity in LMICs is therefore of critical importance. Such a discrepancy could be addressed in part through collaborations between HIC and LMIC researchers and access to international funding bodies,^5^ alongside partnerships between local medical and educational institutions and cleft providers.

Based on the findings of this review, priority areas for future research in LMICs include family functioning and healthcare experiences. Intervening early to improve psychological functioning in caregivers and families from the start of their journey can positively impact the long-term well-being of both the family and the child, the importance of which cannot be underestimated.^10^ Healthcare satisfaction is also a consistent predictor of caregiver well-being,^10^ emphasizing the need to examine how to best deliver high-quality comprehensive cleft care to enhance psychological adjustment. In addition, an increase in our understanding of the self-reported experiences and support needs of children, adolescents, and adults is necessary to make certain these voices are incorporated into the healthcare journey. Where individuals with CL/P are the focus, studies should distinguish between developmentally relevant age groups (eg, infancy = 0-1 year, toddler = 2-3 years, early childhood = 4-6 years, middle childhood = 7-12 years, adolescence = 13-17 years, emerging adulthood = 18-24 years, early adulthood = 25-44 years, middle adulthood = 45-64 years, late adulthood = 65+ years). Additional studies of the role of spirituality and religion may be warranted, and more comprehensive documentation of demographic variables such as socioeconomic status would aid interpretation. Only two interventions were described in the current review,^55,68^ but both reported promising results. Feasibility and acceptability testing of potential interventions is a future step for researchers to embark upon. Future research should also endeavor to include individuals with syndromic CL/P and related conditions to ensure a greater understanding of the spectrum of the condition.^112^

A relatively high proportion of studies included in this review used a qualitative approach, although few involved in-depth analyses. The strength of qualitative methods lies in their ability to elicit detailed accounts from individuals and caregivers and tap into important healthcare issues that would otherwise have been overlooked.^113^ The customary use of qualitative methods as complementary research tools to enhance the evidence base^113^ in LMICs is therefore encouraged. Another key focus for future research is the consistent use of standardized outcome measures, alongside the use of control groups and/or general population normative data. Consensus regarding which measures to use at which time points is an important consideration, as is the translation of these measures, and the collection of country/region-specific norms for relevant interpretation of scores. Data collection from both individuals and their caregivers is important, since agreement ratings can be low, as is coordinated efforts to acquire data over multiple sites. Cross-discipline research could also provide further insight (eg, the role of malnutrition on psychological and developmental outcomes).

A more fundamental research challenge in LMICs is that LMICs are not uniform. Countries and regions have important healthcare, sociocultural, educational, and economic differences that need to be considered. This review did not group specific findings according to geographical region, LMIC classification, or local cultural context. However, it was clear that far fewer studies have been conducted in some geographical regions in comparison to others and that few studies have focused on low-income countries when compared to other LMIC classifications. Further, only 28 of the 135 countries (20.7%) listed as LMICs on the World Bank website in 2024 are currently represented in the psychosocial literature. This review was also only able to include work that has been published in academic journals, a privilege that not all stakeholders have. Researchers could consider working with local communities to document the various initiatives and interventions that are currently being delivered, yet not widely reported. Some eligible studies may also have been omitted during the search process due to international differences in article indexing. Recommendations for future research are provided in Table 3.

## Conclusions

This review has synthesized published literature exploring the psychosocial impact of CL/P in LMICs and set recommendations for future collaborative research and clinical practice. Global health initiatives recognize the right for every child to thrive and emphasize the need for improved health systems to care for children with congenital conditions; enhanced data quality, analysis and application; and increased emotional and social support for affected children and families (WHO, 2023). Commitment to long-term partnerships between NGOs, researchers, clinicians, and local communities is essential to achieve these goals and for the successful implementation of truly comprehensive cleft care.

## Supplemental Material

sj-docx-1-cpc-10.1177_10556656251343393 - Supplemental material for The Psychosocial Impact of Cleft Lip and/or Palate on Caregivers and Individuals in Low- and Middle-Income Countries: A Narrative Review Supplemental material, sj-docx-1-cpc-10.1177_10556656251343393 for The Psychosocial Impact of Cleft Lip and/or Palate on Caregivers and Individuals in Low- and Middle-Income Countries: A Narrative Review by Nicola M. Stock, Bruna Costa, Anna Zarola, Allyn Auslander, Hugh Brewster, Phyllida Swift, Leela Imam, Usama Hamdan, Gareth Davies, Sara Horne and Priti P. Desai in The Cleft Palate Craniofacial Journal
