# Supplementary material for: The Psychosocial Impact of Cleft Lip and/or Palate on Caregivers and Individuals in Low- and Middle-Income Countries: A Narrative Review
Source: Cleft Palate Craniofac J. 2025 Jun 16;63(6):1804–28. doi: 10.1177/10556656251343393 (PMC13176505; doi:10.1177/10556656251343393)
Supplement: sj-docx-1-cpc-10.1177_10556656251343393 - Supplemental material for The Psychosocial Impact of Cleft Lip and/or Palate on Caregivers and Individuals in Low- and Middle-Income Countries: A Narrative Review [file sj-docx-1-cpc-10.1177_10556656251343393.docx]

**Supplemental Table 1:** Search terms used in this review

| **Diagnostic** | Craniofac* OR Cleft lip and palate OR Cleft* OR CLP |
| --- | --- |
| **Population** | Parent* OR Maternal OR Paternal OR Mum OR Mom OR Mother OR Dad OR Father OR Famil* OR Caregiver OR Carer OR Child* OR Adolesc* OR Teen* OR Adult OR Individual OR Person OR People OR Patient |
| **Country** | Low and middle income OR Albania OR Algeria OR Antigua and Barbuda OR Antigua OR Barbuda OR Argentina OR Azerbaijan OR Belarus OR Bosnia and Herzegovina OR Bosnia OR Herzegovina OR Botswana OR Brazil OR People’s Republic of China OR China OR Colombia OR Costa Rica OR Cuba OR Dominica OR Dominican Republic OR Ecuador OR Equatorial Guinea OR Fiji OR Gabon OR Grenada OR Guyana OR Iran OR Iraq OR Jamaica OR Kazakhstan OR Lebanon OR Libya OR Malaysia OR Maldives OR Marshall Islands OR Mauritius OR Mexico OR Montenegro OR Montserrat OR Namibia OR Nauru OR Niue OR North Macedonia OR Palau OR Panama OR Paraguay OR Peru OR Saint Helena OR Saint Lucia OR Saint Vincent and the Grenadines OR Saint Vincent OR The Grenadines OR Samoa OR Serbia OR South Africa OR Suriname OR Thailand OR Tonga OR Turkey OR Turkmenistan OR Venezuela OR Wallis and Futuna OR Wallis OR Futuna OR Afghanistan OR Angola OR Bangladesh OR Benin OR Bhutan OR Burkina Faso OR Burundi OR Cambodia OR Central African Republic OR Chad OR Comoros OR Democratic Republic of the Congo OR Congo OR Djibouti OR Eritrea OR Ethiopia OR Gambia OR Guinea OR Guinea-Bissau OR Haiti OR Kiribati OR Lao People’s Democratic Republic OR Lao OR Lesotho OR Liberia OR Madagascar OR Malawi OR Mali OR Mauritania OR Mozambique OR Myanmar OR Nepal OR Niger OR Rwanda OR Sao Tome and Principe OR Senegal OR Sierra Leone OR Solomon Islands OR Somalia OR South Sudan OR Sudan OR Tanzania OR Timor-Leste OR Togo OR Tuvalu OR Uganda OR Vanuatu OR Yemen OR Zambia OR Democratic People’s Republic of Korea OR Korea OR Zimbabwe OR Armenia OR Bolivia OR Cabo Verde OR Cape Verde OR Cameroon OR Congo OR Cote d’Ivoire OR Egypt OR El Salvador OR Eswatini OR Georgia OR Ghana OR Guatemala OR Honduras OR India OR Indonesia OR Jordan OR Kenya OR Kosovo OR Kyrgyzstan OR Micronesia OR Moldova OR Mongolia OR Morocco OR Nicaragua OR Nigeria OR Pakistan OR Papua New Guinea OR Philippines OR Sri Lanka OR Syrian Arab Republic OR Syria OR Tajikistan OR Tokelau OR Tunisia OR Ukraine OR Uzbekistan OR Vietnam OR West Bank and Gaza Strip |
| **Psychosocial** | Cognit* OR Learn* OR School OR Achieve* OR Language OR Memory OR Intelligen* OR IQ OR Develop* OR Speech OR Health OR Behavio* OR Attention OR Hyperactivity OR Internal* OR External* OR Psych* OR Impact OR Effect OR Adjust* OR Experience OR Emotion* OR Affect OR Stress OR Depress* OR Anx* OR Stigma OR Cop* OR Distress OR Quality of life OR QoL OR Self* OR Resilien* OR Social OR Peer OR Friend* OR Intima* OR Attach* OR Treatment OR Surg* OR Pathway OR Satisfaction OR Function OR Rating OR Outcome OR Body Image OR Appearance |
